# Characterization of a Flavonoid 3’/5’/7-*O*-Methyltransferase from *Citrus reticulata* and Evaluation of the In Vitro Cytotoxicity of Its Methylated Products

**DOI:** 10.3390/molecules25040858

**Published:** 2020-02-15

**Authors:** Xiaojuan Liu, Yue Wang, Yezhi Chen, Shuting Xu, Qin Gong, Chenning Zhao, Jinping Cao, Chongde Sun

**Affiliations:** 1 College of Agriculture & Biotechnology, Zhejiang University, Zijingang Campus, Hangzhou 310058, China; 11416048@zju.edu.cn (X.L.); fruit@zju.edu.cn (Y.W.); yzchen000@163.com (Y.C.); shutingxu@zju.edu.cn (S.X.); 21816138@zju.edu.cn (Q.G.); 11616041@zju.edu.cn (C.Z.); 0017165@zju.edu.cn (J.C.); 2 Zhejiang Provincial Key Laboratory of Horticultural Plant Integrative Biology, Zhejiang University, Zijingang Campus, Hangzhou 310058, China; 3 The State Agriculture Ministry Laboratory of Horticultural Plant Growth, Development and Quality Improvement, Zhejiang University, Zijingang Campus, Hangzhou 310058, China

**Keywords:** citrus, flavonoids, *O*-methyltransferase, bioactivity, cytotoxicity

## Abstract

*O*-methylation of flavonoids is an important modification reaction that occurs in plants. *O*-methylation contributes to the structural diversity of flavonoids, which have several biological and pharmacological functions. In this study, an *O*-methyltransferase gene (*CrOMT2*) was isolated from the fruit peel of *Citrus reticulata*, which encoding a multifunctional *O*-methyltransferase and could effectively catalyze the methylation of 3’-, 5’-, and 7-OH of flavonoids with vicinal hydroxyl substitutions. Substrate preference assays indicated that this recombinant enzyme favored polymethoxylated flavones (PMF)-type substrates in vitro, thereby providing biochemical evidence for the potential role of the enzyme in plants. Additionally, the cytotoxicity of the methylated products from the enzymatic catalytic reaction was evaluated in vitro using human gastric cell lines SGC-7901 and BGC-823. The results showed that the in vitro cytotoxicity of the flavonoids with the unsaturated C2-C3 bond was increased after being methylated at position 3’. These combined results provide biochemical insight regarding CrOMT2 in vitro and indicate the in vitro cytotoxicity of the products methylated by its catalytic reaction.

## 1. Introduction

Flavonoids are secondary metabolites widespread in plants, and are considered to be important small molecules participating in various metabolic pathways. Flavonoids are compounds with C6-C3-C6 skeletons. Based on the saturation of C2-C3 bond and the presence or absence of the hydroxy group at C3, flavonoids can be further divided into several types, including flavones, flavonols, dihydroflavones, and dihydroflavonols [1]. It has been well documented that flavonoids play important roles in plant biology [1,2,3,4,5,6]. For example, flavonoids can form specific pigments to serve as pollinator attractants, help in seed germination, and act as phytoalexins, signal molecules, allopathic compounds, and antimicrobials in plants [4]. Flavonoids can protect plants against various types of damage including that induced by ultraviolet (UV) irradiation, freezing, drought, and nitrogen deficiency [1]. Flavonoids are also thought to be important dietary ingredients and have also been intensively studied for their health benefits to humans and animals [7,8]. Flavonoids produced by plants have diverse biological activities, including anti-inflammatory [9], antioxidant [10], and anti-cancer activities [11].

The functional diversity of flavonoids is based on their structural diversity. It has been estimated that there are more than 10,000 plant-derived flavonoids with diverse structures [12]. The structural diversity of flavonoids is achieved by a number of modifications, including hydroxylation, methylation, glycosylation, and malonylation [12]. In plants, flavonoid *O*-methylation on the hydroxy groups enhances the chemical stability and transportability of flavonoids, and is thus one of the most important modifications [13]. It has also been suggested that methylated flavonoids possess higher biological activity than unmethylated compounds [13]. *O*-methyltransferases (OMTs) can transfer the methyl group to specific hydroxy moieties of flavonoids by utilizing S-Adenosyl-l-methionine (SAM) as a methyl donor; thereby, the regioselective *O*-methylation of flavonoids can be catalyzed. Plant OMTs are a large family; based on their bivalent ion dependency and molecular mass, they are mainly divided into two categories: caffeic acid OMTs (COMTs) and caffeoyl-CoA OMTs (CCoAOMTs) [14]. Both categories of OMTs have been demonstrated to be involved in the methylation of flavonoids [13].

3’-OMTs responsible for 3’-*O*-methylation of flavonoids are the most common flavonoid *O*-methyltransferases (FOMTs) [12]. The 3’-OMTs from different species exhibit distinct catalytic properties. For example, OsOMT1 from *Oryza sativa* [15] and SlMOMT4 from *Solanum lycopersicum* [16] display stringent specificity toward 3’-OH of flavonoids, while other plant 3’-OMTs show broader methylation sites not restricted to the 3’-OH of the B-ring. The following examples are all methylated at both the 3’ and 5’ hydroxy groups of flavonoids: ZmOMT2 from *Zea mays* and HvOMT1 from *Hordeum vulgare* [17], ShMOMT1 from *Solanum habrochaites* [18], CrOMT2 from *Catharanthus roseus* [19], and SlOMT3 from *Solanum lycopersicum* [20]. In particular, TaOMT1 [21] and TaOMT2 [22] from *Triticum aestivum* are both active with all of the hydroxy groups on the B-ring, and can sequentially methylate the 3’, 4’, 5’-OH of tricetin. Published evidence shows that 3’-OMTs from different species display diverse regioselectivity and specificity, indicating that unique 3’-OMTs may exist in species that are rich in various methylated flavonoids, such as citrus. The peel of citrus fruit is also known to accumulate abundant methylated flavonoids, especially polymethoxylated flavones (PMFs) [23].

In silico analysis of the *OMT* family has been comprehensively performed in *Citrus sinensis*, and candidate genes for flavonoid *O*-methylation have been predicted based on the phylogenetic tree [24]. CdFOMT5 from *Citrus depressa* has been demonstrated to be a multifunctional OMT and can methylate the 3-, 5-, 6-, and 7-OH of flavones by in vitro experiment [25]. Cell-free extracts of citrus tissues exhibit 3’-*O*-methylation against several flavonoids containing 3’-OH and 4’-OH, such as quercetin, luteolin, and quercetagetin [26,27]. However, the 3’-OMT responsible for this enzymatic reaction has not been experimentally identified in citrus.

In this study, we isolated and characterized a *3’-OMT* from *Citrus reticulata*, termed *C. reticulata* O-methyltransferase gene (*CrOMT2*). The recombinant CrOMT2 was found to be a 3’/5’/7-OMT by in vitro experiments. To evaluate the potential role of this enzyme in citrus, an analysis was performed for the substrate preference of the recombinant enzyme in combination with the phylogeny. Additionally, flavonoid substrates and their corresponding methylated products were evaluated for their in vitro cytotoxicity activity on two human gastric cell lines, SGC-7901 and BGC-823, respectively.

## 2. Results

### 2.1. CrOMT2 Isolation and Bioinformatic Analysis

It has been proposed that the peel of “Ougan” fruit is rich in PMFs, and 3’-*O*-methylation was common in PMFs [23]. Here, the coding gene of a putative 3’-OMT was isolated from the cDNA library of the Ougan fruit peel. Specifically, the amino acid sequence of a flavonoid 3’-OMT (AtOMT1) from *Arabidopsis thaliana* [28] was used as a query in the local blast against the protein database of *Citrus clementina* to screen for the most promising 3’-OMT in citrus. As shown in Supplementary Table S1, Ciclev10020814m was the most identical OMT, with 73.35% identity to AtOMT1. Based on the *Citrus clementina* genome, gene-specific primers were designed to amplify the coding region of *Ciclev10020814m*. A 1101 bp DNA sequence was then yielded with 98.79% identity to its template DNA sequence (1074 bp) (Supplementary Figure S1). The deduced amino acid sequence of the amplified gene was identical to that of a putative caffeic acid *O*-methyltransferase (GenBank: ADK97702.1) isolated from *Citrus aurantium*, which is a sequence directly submitted to NCBI, without experimental study. Only four nucleotides were not identical between the coding sequences of the two genes (Supplementary Figure S1). The cloned *OMT* in our research was termed *CrOMT2*. It encoded 366 amino acid residues, which can in theory generate a protein with the molecular mass of 39.99 kDa (ExPASy Compute, https://web.expasy.org/compute_pi/). It also included two conserved domains, Methyltranferase_2 (pfam00891) and dimerization (pfam08100). This in silico analysis together indicated that CrOMT2 belongs to the COMT family and is a candidate flavonoid 3’-OMT.

### 2.2. Substrate Specificity and Kinetic Parameters of CrOMT2

The *CrOMT2* gene was expressed by infusing with two histidine tags in *Escherichia coli*, and the theoretically calculated molecular weight for the recombinant enzyme was 58.75 kDa. SDS-PAGE analysis showed that the recombinant enzyme was successfully purified, with its molecular weight consistent with the theoretical one (Supplementary Figure S2). The pH and temperature dependency assays showed that the purified CrOMT2 had high activity at a broad pH range from 8 to 9 (in Tris-HCl buffer), indicating that it preferred an alkaline environment to a neutral or acidic one. The optimum temperature for CrOMT2 was 55 °C and 87.9% activity was lost at 25 °C (Supplementary Figure S2). The following tests were carried out at pH 8.0 and 37 °C to balance the substrate stability and enzyme activity.

**Figure 1:**
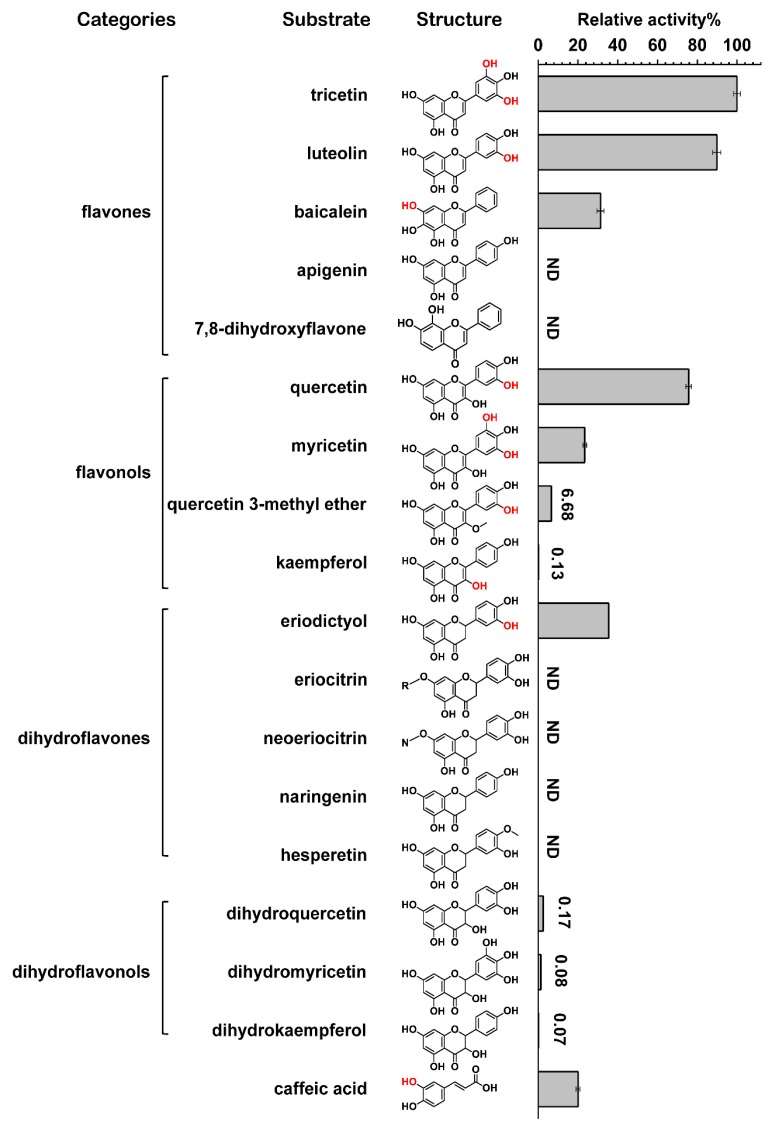
Relative activity of *Citrus reticulata O*-methyltransferase 2 (CrOMT2) against four representative flavonoids and caffeic acid in vitro. The recombinant CrOMT2 purified from *Escherichia coli* was incubated with a number of phenolic substrates, and the relative activity against different substrates was estimated. The methylation sites identified for substrates are indicated in red in their structural formula. ND represents “not detectable”. R- in eriocitrin represents rutinoside and *N*- in neoeriocitrin represents neohesperidoside. The data are shown as means ± SE (n = 3).

To screen the substrate specificity of the purified recombinant CrOMT2, caffeic acid and four representative classes of flavonoids were tested using SAM as the methyl donor (Figure 1). The results of relative activity assays showed that the purified enzyme could methylate a wide array of catechol-type flavonoids except for 7,8-dihydroxyflavone and eriodictyol glycosides (eriocitrin and neoeriocitrin) (Figure 1). Flavonoids without vicinal hydroxy groups were not converted (e.g., apigenin, naringenin, hesperetin) or were only slightly converted (e.g., kaempferol). It could also be concluded that flavones, flavonols, and dihydroflavones were preferred categories of the recombinant CrOMT2 compared with dihydroflavonols (Figure 1). The purified CrOMT2 could also methylate phenylpropanoid caffeic acid with lower relative activity.

**Figure 2:**
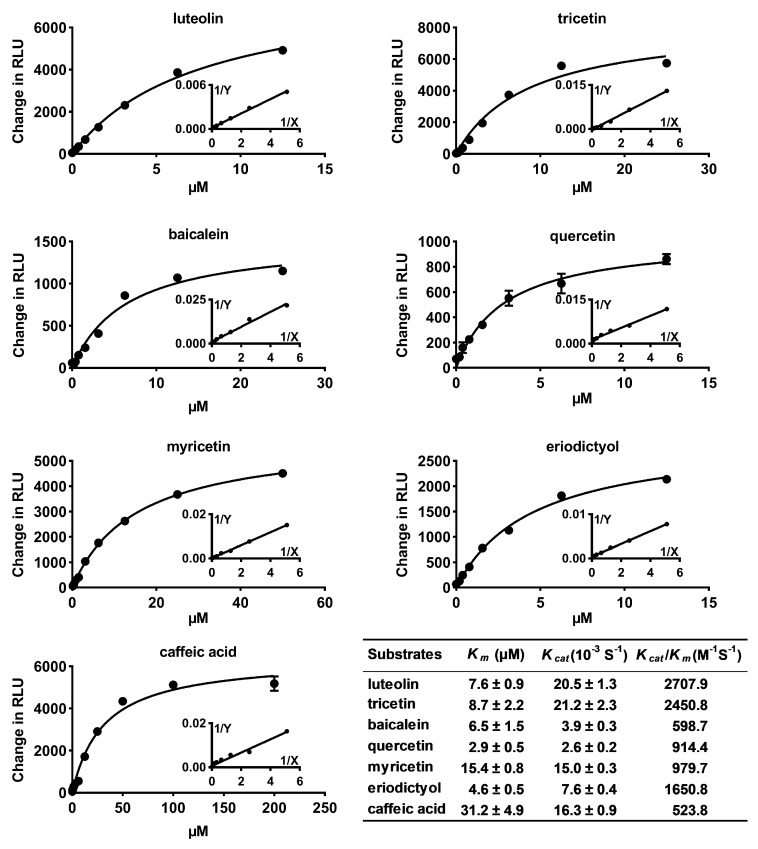
Kinetic analysis of *Citrus reticulata O*-methyltransferase 2 (CrOMT2) using seven substrates. Seven substrates with relative higher activity were selected for kinetic analysis. Apparent *K_m_* and *K_cat_* were estimated using non-linear fit to Michaelis–Menten. Change in RLU (relative light units) indicates background-subtracted RLU. The data are shown as means ± SE (n = 3).

Substrates with relative activity over 7% were used to further investigate the kinetic properties of the recombinant CrOMT2 (Figure 2). The results indicated that the enzyme displayed the highest catalytic efficiencies (*K_cat_*/*K_m_* value: 2707.9 and 2450.8 M^−1^S^−1^) against luteolin and tricetin (two flavones), and had a high affinity for both substrates (*K_m_* values: 7.6 μM and 8.7 μM). Flavone baicalein was also methylated by the enzyme with a similar affinity constant to luteolin and tricetin, but a *K_cat_* value was ca. 5-fold lower than that of these two flavones. The enzyme had a much higher affinity for quercetin than for myricetin. However, due to the much higher turnover rates of the enzyme with myricetin, the catalytic efficiencies for these two flavonols were similar. Dihydroflavone eriodictyol was methylated by the protein with a relatively high catalytic efficiency (*K_cat_*/*K_m_* value 1650.8 M^−1^S^−1^) and an affinity constant of 4.6 μM. The enzyme could also methylate the simple phenolic acid caffeic acid, as a substrate, albeit with a lower affinity (Figure 2).

### 2.3. Identification of Methylated Products

**Figure 3:**
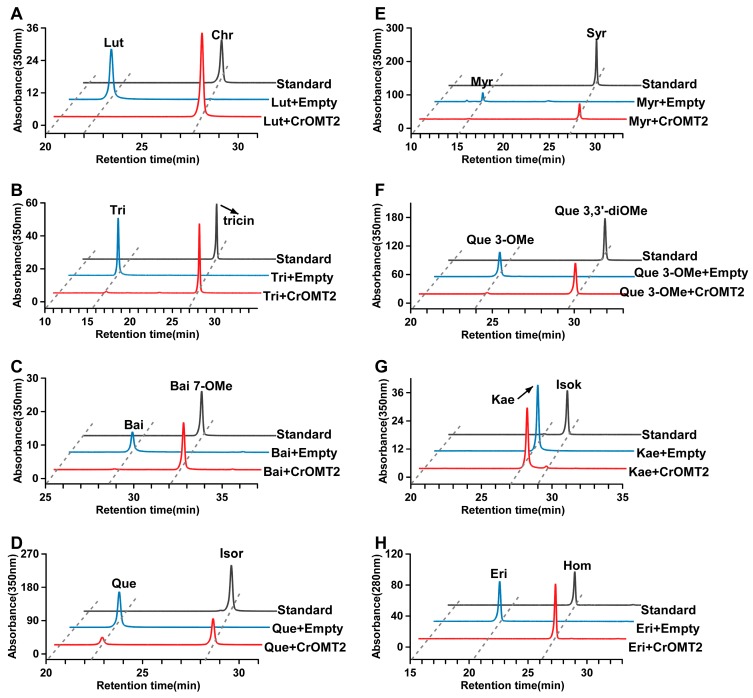
HPLC analysis of products catalyzed by *Citrus reticulata O*-methyltransferase 2 (CrOMT2). The methylated products produced by the recombinant CrOMT2 were identified by comparison of the retention times and MS/MS data with corresponding standards (see Supplementary Figure S3 for the MS/MS data of standards). HPLC chromatograms of enzyme reactions with luteolin (**A**), tricetin (**B**), baicalein (**C**), quercetin (**D**), myricetin (**E**), quercetin 3-methyl ether (**F**), kaempferol (**G**), and eriodictyol (**H**) are indicated. Substrates catalyzed by CrOMT2 indicated in red; substrates incubated with empty vector (pET32a) indicated in blue, commercial standards of the methylated products indicated in black. Lut: luteolin; Chr: chrysoeriol; Tri: tricetin; Bai: baicalein; Bai 7-OMe: baicalein 7-methyl ether; Que: quercetin; Isor: isorhamnetin; Myr: myricetin; Syr: syringetin; Que 3-OMe: quercetin 3-methyl ether; Que 3,3’-diOMe: quercetin 3,3’-dimethyl ether; Kae: kaempferol; Isok: isokaempferide; Eri: eriodictyol; Hom: homoeriodictyol.

**Figure 4:**
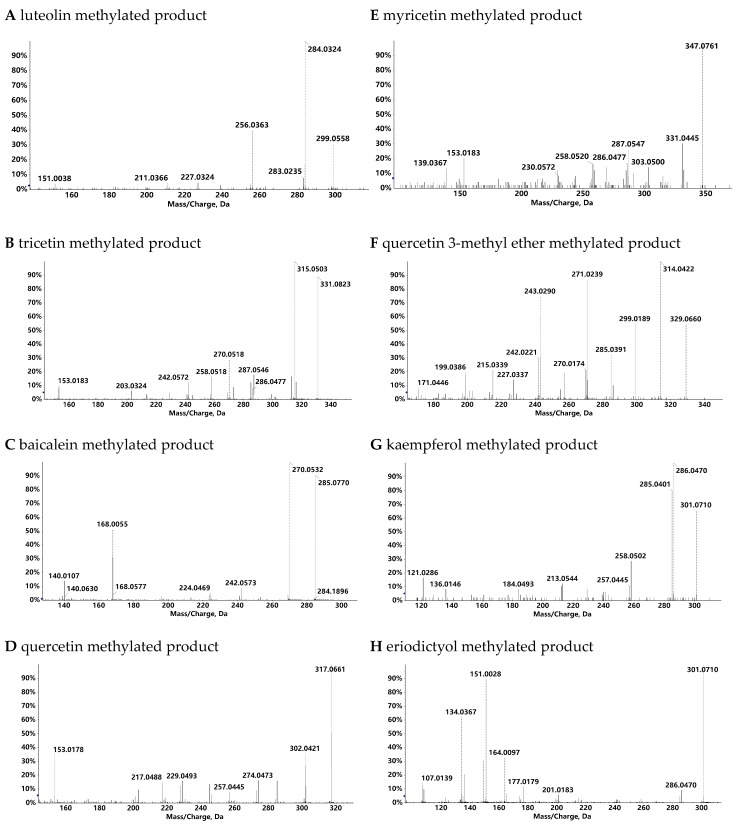
MS/MS spectra of the methylated products catalyzed by *Citrus reticulata O*-methyltransferase 2 (CrOMT2). Substrates utilized were luteolin (**A**), tricetin (**B**), baicalein (**C**), quercetin (**D**), myricetin (**E**), quercetin 3-methyl ether (**F**), kaempferol (**G**), and eriodictyol (**H**). (**A**, **F**, **H**) were operated in negative ion mode and the rest were in positive ion mode. The y axis represents the percent intensity.

The methylated products generated by the recombinant CrOMT2 were identified by comparing the retention times and MS/MS data with the corresponding commercial standards (Figure 3, Figure 4, and Supplementary Figure S3). The enzyme could convert luteolin and tricetin into their corresponding 3’-methyl ether (chrysoeriol) and 3’,5’-dimethyl ether (tricin), respectively (Figure 3A,B). Baicalein, (i.e., 6,7,8-dihydroxyflavone) was methylated at 7-OH, yielding baicalein 7-methyl ether (Figure 3C). Quercetin and myricetin exhibited the same methylation pattern as luteolin and tricetin, and their corresponding 3’-methyl ether (isorhamnetin) and 3’,5’-dimethyl ether (syringetin) were produced by the enzyme, respectively (Figure 3D,E). When quercetin 3-methyl ether served as the substrate, its 3’-*O*-methylation product (quercetin 3,3’-dimethyl ether) was obtained (Figure 3F). The enzyme also displayed a negligible activity to kaempferol, yielding its 3-methylated product (isokaempferide) (Figure 3G). With eriodictyol as a substrate, its 3’-methylation product (homoeriodictyol) was identified (Figure 3H). The recombinant CrOMT2 could also separately methylate the three dihydroflavonols (dihydroquercetin, dihydromyricetin, and dihydrokaempferol) into a single product based on the HPLC chromatogram (Supplementary Figure S4); however, the structures of the methylated products remained to be verified. As a simple phenolic acid, caffeic acid was also converted into ferulic acid (caffeic acid 3-methyl ether), as determined by comparing the retention time of the methylated product with the commercial standard (Supplementary Figure S4).

### 2.4. Phylogenetic Relationship of CrOMT2 to other COMTs

**Figure 5:**
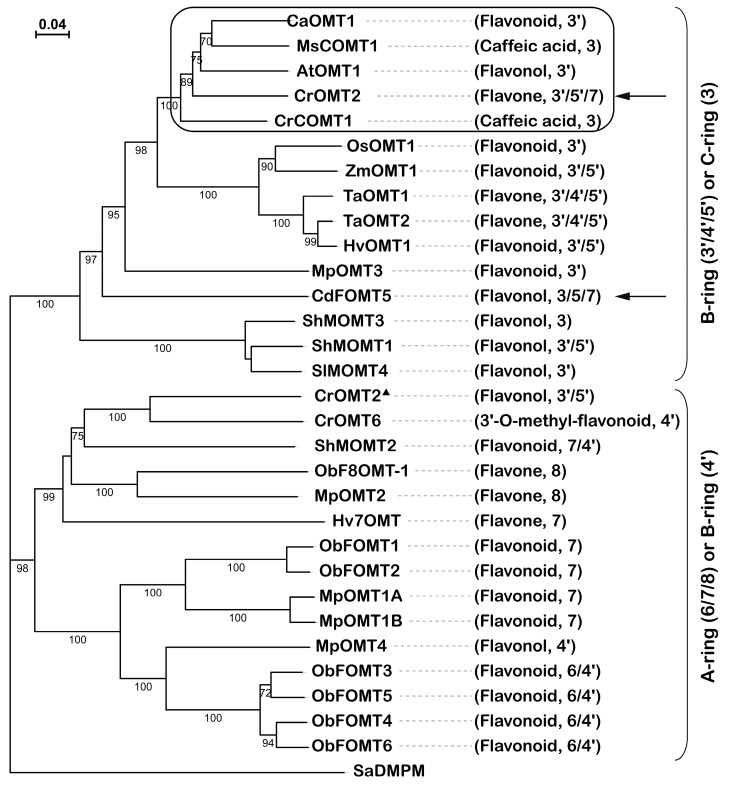
Phylogenetic tree of *Citrus reticulata O*-methyltransferase 2 (CrOMT2) and selected plant caffeic acid *O*-methyltransferases (COMTs) at the protein level. The preferred substrates and methylation positions are indicated for each protein. Percent bootstrap values (n = 1000) greater than 70 are shown in the middle of each branch. The evolutionary history was analyzed using the neighbor-joining method with SaDMPM from *Streptomyces alboniger* acting as the outgroup. Two COMTs from citrus are indicated with arrows. The small clade including CrOMT2 from *C. reticulata* is boxed in black. To avoid ambiguity, CrOMT2 from *Catharanthus roseus* was marked with a triangle. See Supplementary Table S3 for UniProt entries and species of the OMTs.

The phylogenetic tree was constructed using the deduced amino acid sequences of CrOMT2 and other COMTs. CrOMT2 was clustered with the clade in which most members catalyze the hydroxy groups on the B-ring (3’/4’/5’) or C-ring of flavonoids (Figure 5). The other clade consisted of OMTs that methylate the hydroxy moieties on the A-ring (6/7/8) or B-ring (4’) except for CrOMT2 from *C. roseus* (Figure 5). In addition to the expected meta-methylation pattern (3’/5’) on the B-ring, the recombinant CrOMT2 also displayed unusual 7-OH methylation on baicalein. To the best of our knowledge, this is the first report of a 3’/5’ flavonoid *O*-methyltransferase with the capacity of 7-OH methylation. Another unique OMT was CdFOMT5 from *C. depressa*, which also exhibited 7-OH methylation [25] (Figure 5). The residues associated with the catalytic function of CrOMT2 were also inferred according to previous studies [29] (Supplementary Figure S5). It could be indicated that the residues and motifs (indicated with red asterisks) responsible for SAM binding were conserved across the aligned COMTs.

### 2.5. In Vitro Cytotoxicity of Flavonoids on Human Gastric Cancer Cells

Given the diverse methylation sites of the recombinant CrOMT2 against different flavonoids, six substrates and their corresponding methylation products were used to assess their cytotoxicity against human gastric cancer cells in vitro. Two human gastric cancer cell lines, i.e., SGC-7901 and BGC-823, were used for the cytotoxicity assay. The flavonoid concentration gradients were set in a range from 3.13 μM to 50 μM (Supplementary Figure S6). Results showed that two products catalyzed by CrOMT2 produced stronger inhibitory effects on the human gastric cancer cells compared to luteolin and quercetin substrates in vitro (Table 1). Specifically, the inhibitory effects of chrysoeriol on SGC-7901 and BGC-823 cells were 1.15- and 1.68-fold higher than those of luteolin, respectively, while the inhibitory effects of isorhamnetin on SGC-7901 and BGC-823 were 3.54- and 6.68-fold higher than those of quercetin, respectively. As for the substrates with improved activities after methylation, their structures were characterized by the double bond at the C-ring and the methylation site of the B-ring toward the 3’ position compared to the flavonoids with no increase in activities. Paclitaxel was used as a positive control, and its IC_50_ values for SCG-7901 and BGC-823 cells were 0.06 and 0.07 μM, respectively (data not shown). DMF was used as a solvent control and showed no inhibitory effect on the two cell lines at the tested concentration.

## 3. Discussion

In this study, the coding gene of a putative flavonoid 3’-*O*-methyltransferase, named *CrOMT2*, was isolated from citrus and successfully expressed in vitro in *E. coli*. The purified CrOMT2 could methylate a number of flavonoids with vicinal hydroxy groups in vitro, with the methylation sites occurring at 3’-OH, 5’-OH, or 7-OH. The inhibitory effect of the specific methylation on human gastric cancer cells was examined. The in vitro cytotoxicity of the two substrates (i.e., luteolin and quercetin) showed a significant increase after being methylated at 3’-OH.

The recombinant CrOMT2 could methylate numerous flavonoids with vicinal B-ring hydroxy moieties in vitro; this finding was consistent with the catalytic activity of cell-free extract from citrus tissues [27]. This substrate specificity was also similar to AtOMT1 from *A. thaliana* [28], which clustered closely to CrOMT2 on the phylogenetic tree (Figure 5). Two flavones, i.e., luteolin and tricetin, were the most preferred substrates for CrOMT2 among other tested substrates (Figure 1 and Figure 2). This strong preference to the two flavones also occurred in other COMTs: TaOMT1 [21] and TaOMT2 [22] from *T. aestivum*, ZmOMT2 from *Z. mays*, and HvOMT1 from *H. vulgare* [17]. In contrast to CrOMT2, which catalyzed luteolin as effectively as tricetin (Figure 2), all of the above four OMTs exhibited a preference to tricetin over luteolin as a substrate. As these four OMTs effectively converted tricetin into its 3’,5’-dimethyl ether (tricin), they were considered to be involved in the synthesis of tricin, a characteristic flavone of cereal grain plants [17,21,22]. As a flavone with three hydroxy groups at the A-ring, baicalein was also methylated by the recombinant CrOMT2, with a lower turnover rate compared to the luteolin and tricetin (Figure 2). Thus, CrOMT2 might be involved in the synthesis of PMFs in citrus due to its preference for flavones, which were exactly the PMF-type substrates [23]. In contrast to many other FOMTs capable of 3’-methylation with lower [17,20,30] or no activities [28,31] on eriodictyol (a dihydroflavone), CrOMT2 showed high affinity and catalytic efficiency with this substrate and produced its 3’-methyl ether derivative, homoeriodictyol (Figure 2 and Figure 3H). Given the ubiquitous eriodictyol glycosides in citrus [23], CrOMT2 was determined a promising enzyme involved in the homoeriodictyol synthesis in vivo. As two widespread flavonols in plants [32], quercetin and myricetin, were also methylated by the recombinant CrOMT2, their products were confirmed as 3’-methyl quercetin (isorhamnetin) and 3’,5’-dimethyl myricetin (syringetin), respectively (Figure 3D,E). When myricetin serves as a substrate, similarly to CrOMT2, flavonoid 3’-OMTs often sequentially methylate both 3’-OH and 5’-OH due to the similar chemical properties of the hydroxy groups in the two positions [18,19,20]. However, there were several flavonoid 3’-OMTs that only transferred one methyl group into the 3’-OH of myricetin, e.g., OsOMT1 from *O. sativa* [15] and SlMOMT4 from *S. lycopersicum* [16]. Except for the activity toward flavonoids, the purified CrOMT2 also showed relatively lower activity toward caffeic acid (Figure 1), which is an important intermediate compound involved in lignin biosynthesis [33]. This was consistent with many characterized FOMTs belonging to COMTs [15,19,21,28,30,34,35]. CrOMT2 was closely clustered within a small clade in which both typical caffeic acid 3-OMTs and flavonoid 3’-OMTs were located (Figure 5). AtOMT1, sister to CrOMT2 in this clade, has been identified as a flavonol 3’-OMT. This identification was confirmed by the substrate specificity assay in vitro [28] and was further verified by the analysis of flavonoid profile in the mutant *omt1* [36]. Moreover, it was reported that AtOMT1 was involved in the biosynthesis of lignin and soluble sinapate esters [37]. There were two typical caffeic acid 3-OMTs in this clade (Figure 5). One was MsCOMT1 from alfalfa (*Medicago sativa*), which has a strong impact on lignin biosynthesis that has been verified by downregulating its coding gene in transgenic alfalfa [38]. The other was CrCOMT1 from *C. roseus*. It also favored phenylpropanoids compared to flavonoids in vitro, and this substrate preference indicated the potential role of the enzyme in the lignin pathway [39]. The two lignin-related OMTs together with the bifunctional AtOMT1 in this clade indicated that CrOMT2 could participate in the lignin pathway in addition to flavonoid metabolism.

7-*O*-methylation was detected when using baicalein as a substrate for CrOMT2 (Figure 3C). 7-FOMTs usually have stringent specificity and cluster closely in the phylogenetic tree (Figure 5). Several plant 7-FOMTs have been characterized, e.g., ObFOMT1 and ObFOMT2 from *Ocimum basilicum* [40], MpOMT1A and MpOMT1B from *Mentha x piperita* [35], Hv7OMT from *H. vulgare* [41], and OsNOMT from *O. sativa* [34]. Of the B-ring (3’/4’/5’) or C-ring FOMTs, only two citrus OMTs exhibited activity on 7-OH. One is CrOMT2, reported in our study, and the other one is CdFOMT5 from *C. depressa*, which can methylate 3-, 5-, 6-, and 7-hydroxy groups of flavonoids [25] (Figure 5). Cell-free extracts from citrus also showed 7-*O*-methylation on several flavonoids, including baicalein [26,27]. The unique 7-*O*-methylation catalytic activity of citrus OMTs indicated the significance of 7-*O*-methylation in synthesizing PMFs in citrus. It has been reported that the 7-*O*-glycosides of flavonoids (e.g., narirutin, naringin, hesperidin, and neohesperidin) are the most common flavonoid derivatives in citrus [23], whereas PMFs in citrus usually accumulate in the form of free aglycones instead of glycosides [23]. Since the 7-*O*-glycosylation reaction can be completely blocked by the 7-*O*-methylation, the flux toward PMFs aglycones was likely determined by the 7-*O*-methylation. Similarly, in plants that did not produce 3-*O*-glycosylated flavonoids, the 3-OH methylation also appeared to be the first procedure [42]. Further experiments are needed to verify the hypothesis on the methylation order of flavonoids in citrus. Given the high affinity of CrOMT2 towards baicalein (a PMF-type substrate), CrOMT2 remains a promising 7-OMT in citrus.

## 4. Materials and Methods

### 4.1. Materials

Ougan (*Citrus reticulata* cv. *Suavissima*) fruits were randomly collected for the subsequent experiments at 160 days after flowering. The peels of the fruits were ground in liquid nitrogen to isolate RNA. Human gastric cancer cell lines SGC-7901 and BGC-823 were kindly provided by the Second Affiliated Hospital of Zhejiang University.

### 4.2. Chemicals and Reagents

Mobile phases of acetonitrile and methanol for HPLC (high-performance liquid chromatography) and phenolic acids, including caffeic acid, ferulic acid, naringenin, neoeriocitrin, and hesperetin were purchased from Sigma-Aldrich (St. Louis, MO, USA). Dihydroflavonols (dihydroquercetin dihydromyricetin and dihydrokaempferol), eriodictyol, homoeriodictyol, quercetin 3,3’-dimethyl ether, quercetin 3-methyl ether, chrysoeriol, and isokaempferide were obtained from BioBioPha Co., Ltd. (Kunming, China). Eriocitrin, baicalein, luteolin, quercetin, kaempferol, isorhamnetin, and SAM were from Aladdin (Shanghai, China). Apigenin, myricetin, and 7,8-dihydroxyflavone hydrate were from TCI (Shanghai, China) and tricin was purchased from ChromaDex (Irvine, CA, USA). Tricetin and syringetin were purchased from Extrasynthese (Lyon, France) and baicalein 7-methyl ether was purchased from Shanghai yuanye Bio-Technology Co., Ltd. (Shanghai, China). RPMI-1640 medium, fetal bovine serum (FBS), HEPES, and trypsin-EDTA were purchased from Gibco (Waltham, MA, USA). Cell Counting Kit-8 was purchased from Dojindo Technology (Shanghai, China).

### 4.3. Isolation, Cloning, and Heterologous Expression of CrOMT2

The extraction of total RNA from the peel of Ougan fruit was conducted as described by Kiefer et al. [43], and cDNA was synthesized from 1 μg RNA using a PrimeScript™RT Reagent Kit (Takara, Dalian, China). The full coding sequence (CDs) of *CrOMT2* was amplified by PCR. The cDNA from Ougan fruit peel was used as a template and the annealing primers were designed based on the coding sequence of *Ciclev10020814m* on the *Citrus clementina* reference genome (http://www.citrusgenomedb.org/species/clementina/genome1.0). The cloned CDs was further inserted into the pET32a vector and the expression plasmid *CiOMT2*-pET32a was transformed to the *E. coli* BL21(DE3)pLysS cells (Promega, Madison, WI, USA) for protein expression. Bacterial cultures were incubated in Luria–Bertani medium (0.1 g·L^−1^ ampicillin) at 37 °C until OD_600_ reached 0.6. Protein expression was then induced by adding 1 mM isopropyl β-thiogalactopyranoside at 18 °C for 24 h. The induced bacterial cells were lysed by sonication in 1 × PBS buffer, and the supernatants were then subjected to Co^2+^-affinity chromatography to purify recombinant protein, as described in the user manual of HisTALON™ Gravity Columns Purification Kit (Takara, Dalian, China). Eluted fractions were applied to a PD-10 desalting column (GE Healthcare, Uppsala, Sweden) to store the purified protein in a Tris-HCl buffer (50 mM, 10% glycerol, 2 mM dithiothreitol, pH 8.0) at −80 °C. Oligo nucleotides used above are listed in Supplementary Table S2.

### 4.4. Phylogenetic Tree and Protein Alignments

CrOMT2 and several identified COMTs from plant were aligned using ClustalW. The evolutionary history was constructed by MEGA X tool [44] using the neighbor-joining method [45], and the phylogenetic tree was assessed with 1000 bootstrap replicates [46]. Accession numbers of proteins used are given in Supplementary Table S3. The structure-based alignment of the OMTs was inferred by T-Coffee (Expresso) [47] and deciphered via ESpript 3.0 [48].

### 4.5. Enzyme Activity Measurements and Kinetic Analysis

A wide range of flavonoids along with caffeic acid were incubated with recombinant CrOMT2-pET32a to explore the substrate specificity. A final volume of 200 μL was used with 1 mM SAM as methyl donor, 200 μM phenolic substrates, and 25 μL purified enzyme in 50 mM Tris-HCl buffer, pH 8.0. Assays were conducted for 2 h at 37 °C and quenched with 200 μL methanol. The reaction products were filtered through a 0.22 μm filter and were analyzed by HPLC and mass spectrometry as described below.

Tris-HCl buffer (pH 8.0–9.0) and potassium phosphate buffer (pH 5.5–7.5) were used to investigate the effect of pH on CrOMT2 activity. The temperature dependency was assessed between 25 °C and 60 °C.

Relative activity on different substrates was determined using an MTase-Glo™ Methyltransferase Assay kit (Promega, Madison, WI, USA). It should be noted that the demethylated product of SAM, i.e., S-adenosyl homocysteine (SAH), in the reaction was correlated to luminescence detected by a luminometer. Specifically, 12.5 μM substrate, 250 μM SAM, and diluted protein were incubated in Tris-HCl buffer (pH 8.0) at 37 °C for 30 min. The reaction was terminated by adding 0.5% trifluoroacetic acid, and then the luminescence converted by SAH was assessed via a plate-reading luminometer.

The kinetic properties were assessed as described in the method using 2 mM SAM and a set of substrates at different concentrations. The apparent *K_m_* and *K_Cat_* were calculated using a nonlinear regression curve. The calculation was fitted to Michaelis–Menten via GraphPad Prism version 7 (GraphPad Software, San Diego, CA, USA).

### 4.6. Cell Culture and Cell Viability Assay

Two human gastric cancer cell lines, i.e., SGC-7901 and BGC-823, were used in the cell viability assay. These classic cell lines are commonly used to explore the effect of proliferation inhibition of gastric cancer cells [11,23,49]. The two cell lines were cultured in RPMI-1640 medium (10% FBS and 1 × HEPES) and maintained at 37 °C in a humidified incubator containing 5% CO_2_. Cells were passaged using trypsin-EDTA before reaching 90% confluence. A Cell Counting Kit-8 was used to evaluate the cell viability according to the manufacturer’s instructions. Briefly, 4 × 10^3^ cells per well for SGC-7901 and 8 × 10^3^ cells per well for BGC-823 were seeded onto a 96 well plate and cultured overnight. The cells were replenished with the fresh medium the next day and different concentrations of flavonoids dissolved in DMF (*N*,*N*-dimethyl-formamide) were added. The DMF was used as a solvent control with a final concentration of 0.1%. Paclitaxel was used as a positive control. After 48 h incubation, the supernatant was discarded and the cells were washed twice with 1 × PBS. An equal amount of cck-8 solution (dissolved in FBS-free RPMI-1640 medium) was added into the well and cultured for 1 h. The light absorbance values at 450 nm and 620 nm were measured using a microplate reader and the inhibition ratio was calculated as (A_450_−A_620_)/A_450_. IC_50_ values were calculated using SPSS 19.0 software (IBM, Armonk, NY, USA). The cell viabilities under different treatments were compared using the Tukey test in GraphPad Prism version 7 (GraphPad Software, San Diego, CA, USA).

### 4.7. HPLC and MS/MS Analysis

HPLC analysis was performed on an Agilent 1260 HPLC system (Agilent Technologies, Santa Clara, CA, USA) with a Sunfire C18 ODS column (4.6 × 250 mm, 5 μm, Waters Corp., Milford, MA, USA). The mobile phases consisted of acetonitrile (A) and water with 0.1% formic acid (B). The compounds were segregated with linear gradient run as follows: 20% A (0–5 min), 20%–27% A (5–10 min), 27% A (10–15 min), 27%–40% A (15–25 min), 40%–60% A (25–35 min), 60%–80% A (35–40 min), 80%–100% A (40–42 min), 100%–20% A (42–45 min), 20% A (45–50 min). The flow rate was 1 mL·min^−1^ and injection volume was 10 μL. Chromatographic detection was done using a VWD detector at the wavelength of 280 nm for dihydroflavones, dihydroflavonols, and caffeic acid, and at 350 nm for flavones and flavonols.

High-resolution mass spectrometry was conducted with an AB TripleTOF 5600plus System (AB SCIEX, Framingham, MA, USA). MS/MS analysis was carried out in the positive ion mode (ESI) or negative ion mode, and the exact mass was then obtained.

### 4.8. Statistical Analyses

All results were from at least three replicates and expressed as means ± standard errors (SE). Graphics were made with OriginPro 2019b Learning Edition (Microcal Software Inc., Northampton, MA, USA). Structural formulas were generated using ChemBioDraw Ultra 12.0 (PerkinElmer Informatics, Waltham, MA, USA).

## 5. Conclusions

CrOMT2 from citrus was a typical FOMT belonging to COMTs that transferred methyl groups into the 3’/5’/7-hydroxy groups of the flavonoids backbone in vitro. It was found that the enzyme favored PMF-type substrates, and its methylation sites tended to be critical for PMFs synthesis in citrus. This recombinant CrOMT2 also displayed a broad substrate spectrum and various methylation sites in vitro. The 3’-methylation that occurred in flavones or flavonols (having unsaturated C2-C3 bonds) all increased the in vitro cytotoxicity. This was the first evaluation of the effects of citrus enzymes on human health-related biological activities. The implications of this study may provide a new strategy for the directional biosynthesis of bioactive substances.

## Figures and Tables

**Table 1:** Inhibitory effects of flavonoids catalyzed by *Citrus reticulata O*-methyltransferase 2 (CrOMT2) on the human gastric cancer cells in vitro.

	Substrate	IC_50_ (μM)	Product	IC_50_ (μM)
**SCG-7910**	tricetin	78.8	tricin	\
	luteolin	15.1	chrysoeriol	13.3
	baicalein	37.5	baicalein 7-methyl ether	40.2
	quercetin	37.7	isorhamnetin	10.7
	myricetin	\	syringetin	\
	eriodictyol	66.4	homoeriodictyol	166
**BGC-823**	tricetin	\	tricin	\
	luteolin	41.2	chrysoeriol	24.2
	baicalein	86.9	baicalein 7-methyl ether	\
	quercetin	322	isorhamnetin	48.4
	myricetin	\	syringetin	\
	eriodictyol	188	homoeriodictyol	\

## Supplementary Materials

The following are available online. Figure S1: Sequence alignment of CrOMT2 and two paralogous genes from citrus, Figure S2: biochemical characterization of the recombinant CrOMT2, Figure S3: MS/MS spectra of the authentic standards corresponding to the methylated products catalyzed by CrOMT2, Figure S4: HPLC analysis of dihydroflavonols and caffeic acid methylation by recombinant CrOMT2, Figure S5: Sequence alignment of CrOMT2 and other COMTs from plants, Figure S6: Cell viability after treated with flavonoids substrates or methylated products of CrOMT2 in vitro, Table S1: Percent identity of AtOMT1 against OMTs in *Citrus clementina*, Table S2: Primers used for amplification, Table S3: UniProt entries of COMT proteins used in the phylogenetic tree.
