# Supplementary material for: Characterization of a Flavonoid 3’/5’/7-O-Methyltransferase from Citrus reticulata and Evaluation of the In Vitro Cytotoxicity of Its Methylated Products
Source: Molecules. 2020 Feb 15;25(4):858. doi: 10.3390/molecules25040858 (PMC7070609; doi:10.3390/molecules25040858)
Supplement: Supplementary file 1 [file molecules-25-00858-s001.pdf]

Supplementary Materials

Figure S1

A

```
                           *        20         *        40         *        60         *        80         *
Ciclev10020814m : ATGGGTTCAACCAGTTCAGAAACTCAAATAAGTCCAGCCCAAGGCTCGGATGAAGAGGCAAACCTCTTGGCCATGCAATTAACCAGTGCCTCAGT :  95
CrOMT2          : ATGGGTTCAACCAGTTCAGAAACTCAAATAAGTCCAGCCCAAGGCTCGGATGAAGAGGCAAACCTCTTGGCCATGCAATTAACCAGTGCCTCAGT :  95
HM641694.1      : ATGGGTTCAACCAGTTCAGAAACTCAAATAAGTCCAGCCCAAGGCTCGGATGAAGAGGCAAACCTCTTGGCCATGCAATTAACCAGTGCCTCAGT :  95
                  ATGGGTTCAACCAGTTCAGAAACTCAAATAAGTCCAGCCCAAGGCTCGGATGAAGAGGCAAACCTCTTGGCCATGCAATTAACCAGTGCCTCAGT

                     100         *       120         *       140         *       160         *       180         *
Ciclev10020814m : CTTGCCTATGGTTCTCAAATCAGCCATTGAGCTTGATCTTTTAGAGATCATCGCTAAAGCTGGGCCAGATGCTTTCATGTCTCCAAAAGACATAG :  190
CrOMT2          : CTTGCCTATGGTTCTCAAATCAGCCATTGAGCTTGATCTTTTAGAGATCATCGCTAAAGCTGGGCCAGATGCTTTCATGTCTCCAAAAGACATAG :  190
HM641694.1      : CTTGCCTATGGTTCTCAAATCAGCCATTGAGCTTGATCTTTTAGAGATCATCGCTAAAGCTGGGCCCGATGCTTTCATGTCTCCAAAAGACATAG :  190
                  CTTGCCTATGGTTCTCAAATCAGCCATTGAGCTTGATCTTTTAGAGATCATCGCTAAAGCTGGGCCaGATGCTTTCATGTCTCCAAAAGACATAG

                          200         *       220         *       240         *       260         *       280
Ciclev10020814m : CTTCTCAGCTGCCCACAAAGAACCCAGATGCCCATATCGTGCTTGATCGTATATTGCGCCTTCTGGCGAGCTATTCAGTCCTTAATTGCTCTTTG :  285
CrOMT2          : CTTCTCAGCTGCCCACAAAGAACCCAGATGCCCATATCGTGCTTGATCGTATATTGCGCCTTCTGGCGAGCTATTCAGTCCTTAATTGCTCTTTG :  285
HM641694.1      : CTTCTCAGCTGCCCACAAAGAACCCAGATGCCCATATCGTGCTTGATCGTATATTGCGCCTTCTGGCGAGCTATTCAGTCCTTAATTGCTCTTTG :  285
                  CTTCTCAGCTGCCCACAAAGAACCCAGATGCCCATATCGTGCTTGATCGTATATTGCGCCTTCTGGCGAGCTATTCAGTCCTTAATTGCTCTTTG

                     *       300         *       320         *       340         *       360         *       380
Ciclev10020814m : CGCAATCTCCCCGACGGCAAAGTTGAGAGGCTTTATGGCCTTGCCCCCGTTTGTAAATTCCTCACTAAAAATGAAGATGGTGTTACACTTTCCGA :  380
CrOMT2          : CGCAATCTCCCCGACGGCAAAGTTGAGAGGCTTTATGGCCTTGCCCCCGTTTGTAAATTCCTCACTAAAAATGAAGATGGTGTTACACTTTCCGA :  380
HM641694.1      : CGCAATCTCCCCGACGGCAAAGTTGAGAGGCTTTATGGCCTTGCCCCCGTTTGTAAATTCCTCACTAAAAATGAAGATGGTGTTACACTTTCCGA :  380
                  CGCAATCTCCCCGACGGCAAAGTTGAGAGGCTTTATGGCCTTGCCCCCGTTTGTAAATTCCTCACTAAAAATGAAGATGGTGTTACACTTTCCGA

                           *       400         *       420         *       440         *       460         *
Ciclev10020814m : TCTTTGTCTCATGAACCAAGACAAGGTTCTCATGGAGAGCTGGTACTACTTAAAAGATGCAGTGCTTGAAGGTGGCATTCCATTTAACAAGGCCC :  475
CrOMT2          : TCTTTGTCTCATGAACCAAGACAAGGTTCTCATGGAGAGCTGGTACTACTTAAAAGATGCAGTGCTTGAAGGTGGCATTCCATTTAACAAGGCCT :  475
HM641694.1      : TCTTTGTCTCATGAACCAAGACAAGGTTCTCATGGAGAGCTGGTACTACTTAAAAGATGCAGTGCTTGAAGGTGGCATTCCATTTAACAAGGCCT :  475
                  TCTTTGTCTCATGAACCAAGACAAGGTTCTCATGGAGAGCTGGTACTACTTAAAAGATGCAGTGCTTGAAGGTGGCATTCCATTTAACAAGGCCt

                     480         *       500         *       520         *       540         *       560         *
Ciclev10020814m : ATGGGATGAATGCATTCGATTACCATGGCAAAGATCTAAGATTCAACAAGATTTTCAACAATGGAATGTCTTCTCATTCTACCATTACCATGAAG :  570
CrOMT2          : ATGGGATGAATGCATTCGATTACCATGGCAAAGATCTAAGATTCAACAAGATTTTCAACAATGGAATGTCTTCTCATTCTACCATTACCATGAAG :  570
HM641694.1      : ATGGGATGAATGCATTCGATTACCATGGCAAAGATCTAAGATTCAACAAGATTTTCAACAATGGAATGTCTTCTCATTCTACCATTACCATGAAG :  570
                  ATGGGATGAATGCATTCGATTACCATGGCAAAGATCTAAGATTCAACAAGATTTTCAACAATGGAATGTCTTCTCATTCTACCATTACCATGAAG

                          580         *       600         *       620         *       640         *       660
Ciclev10020814m : AAAATTCTTGAAAATTACAAAGGGTTTGAAGGCCTCAACTCAGTTGTCGACGTTGGTGGTGGAATTGGAGCCACACTTAACATGATTATCTCCAA :  665
CrOMT2          : AAAATTCTTGAAAATTACAAAGGGTTTGAAGGCCTCAACTCAGTTGTCGACGTTGGTGGTGGAATTGGAGCCACACTTAACATGATTATCTCCAA :  665
HM641694.1      : AAAATTCTTGAAAATTACAAAGGGTTTGAAGGCCTCAACTCAGTTGTCGACGTTGGTGGTGGAATTGGAGCCACACTTAACATGATTATCTCCAA :  665
                  AAAATTCTTGAAAATTACAAAGGGTTTGAAGGCCTCAACTCAGTTGTCGACGTTGGTGGTGGAATTGGAGCCACACTTAACATGATTATCTCCAA

                     *       680         *       700         *       720         *       740         *       760
Ciclev10020814m : GTATCCATCGATTAAAGGCATCAACTTTGATTTGCCACATGTGTCGAG---------------------------CATGTTAGGGGGAGACATGT :  733
CrOMT2          : GTATCCATCGATTAAAGGCATCAACTTTGATTTGCCACATGTTATTCAGGATGCTCCAGCTTTTCCTGGTGTCGAGCATGTTGGGGGAGACATGT :  760
HM641694.1      : GTATCCATCCATTAAAGGCATTAACTTTGATTTGCCACATGTTATTCAGGATGCTCCAGCTTTTCCTGGTGTCGAGCATGTTGGGGGAGACATGT :  760
                  GTATCCATCgATTAAAGGCATcAACTTTGATTTGCCACATGTtattcaggatgctccagcttttcctggtgtcgagcatgTtGGGGGAGACATGT

                           *       780         *       800         *       820         *       840         *
Ciclev10020814m : TTGTTAGTGTTCCAAAGGGAGATGCCATTTTTATCAAGTGGATATGTCATGATTGGAGTGATGAGCACTGCGTGAAATTCTTGAAGAACTGCTAT :  828
CrOMT2          : TTGTTAGTGTTCCAAAGGGAGATGCCATTTTTATCAAGTGGATATGTCATGATTGGAGTGATGAGCACTGCGTGAAATTCTTGAAGAACTGCTAT :  855
HM641694.1      : TTGTTAGTGTTCCAAAGGGAGATGCCATTTTTATCAAGTGGATATGTCATGATTGGAGTGATGAGCACTGCGTGAAATTCTTGAAGAACTGCTAT :  855
                  TTGTTAGTGTTCCAAAGGGAGATGCCATTTTTATCAAGTGGATATGTCATGATTGGAGTGATGAGCACTGCGTGAAATTCTTGAAGAACTGCTAT

                     860         *       880         *       900         *       920         *       940         *
```

```
Ciclev10020814m : GAAGCACTCCCAGTAAATGGGAAAGTCATTGTTGCTGAATCTATCCTCCCAGTAACCCCGGACACAAGCCTCGCATCCAAAGTAGTCATCCATGT :  923
CrOMT2          : GAAGCACTCCCAGTAAATGGGAAAGTCATTGTTGCTGAATCTATCCTCCCAGTAACCCCGGACACAAGCCTCGCATCCAAAGTAGTCATCCATGT :  950
HM641694.1      : GAAGCACTCCCAGTAAATGGGAAAGTCATTGTTGCTGAATCTATCCTCCCAGTAACCCCGGACACAAGCCTTGCATCCAAAGTAGTCATCCATGT :  950
                  GAAGCACTCCCAGTAAATGGGAAAGTCATTGTTGCTGAATCTATCCTCCCAGTAACCCCGGACACAAGCCTcGCATCCAAAGTAGTCATCCATGT

                          960         *         980         *        1000         *        1020         *        1040
Ciclev10020814m : CGACTGCATCATGTTGGCTCATAACCCGGGTGGCAAAGAGAGGACTGAACAAGAGTTCAGAGCATTGGCTAAGGCTGCTGGATTCCAAGGTTTCC : 1018
CrOMT2          : CGACTGCATCATGTTGGCTCATAACCCGGGTGGCAAAGAGAGGACTGAACAAGAGTTCAGAGCATTGGCTAAGGCTGCTGGATTCCAAGGTTTCC : 1045
HM641694.1      : CGACTGCATCATGTTGGCTCATAACCCGGGTGGCAAAGAGAGGACTGAACAAGAGTTCAGAGCATTGGCTAAGGCTGCTGGATTCCAAGGTTTCC : 1045
                  CGACTGCATCATGTTGGCTCATAACCCGGGTGGCAAAGAGAGGACTGAACAAGAGTTCAGAGCATTGGCTAAGGCTGCTGGATTCCAAGGTTTCC

                      *        1060         *        1080         *        1100
Ciclev10020814m : AAGTTGTGAGCTCTGCTTTTAATACTTACATTATGGAATTTCTCAAGAGTGCTTGA : 1074
CrOMT2          : AAGTTGTGAGCTCTGCTTTTAATACTTACATTATGGAATTTCTCAAGAGTGCTTGA : 1101
HM641694.1      : AAGTTGTGAGCTCTGCTTTTAATACTTACATTATGGAATTTCTCAAGAGTGCTTGA : 1101
                  AAGTTGTGAGCTCTGCTTTTAATACTTACATTATGGAATTTCTCAAGAGTGCTTGA
```

B

```
                          *          20         *          40         *          60         *          80         *
Ciclev10020814m : MGSTSSETQISPAQGSDEEANLLAMQLTSASVLPMVLKSAIELDLLEIIAKAGPDAFMSPKDIASQLPTKNPDAHIVLDRILRLLASYSVLNCSL :  95
CrOMT2          : MGSTSSETQISPAQGSDEEANLLAMQLTSASVLPMVLKSAIELDLLEIIAKAGPDAFMSPKDIASQLPTKNPDAHIVLDRILRLLASYSVLNCSL :  95
ADK97702.1      : MGSTSSETQISPAQGSDEEANLLAMQLTSASVLPMVLKSAIELDLLEIIAKAGPDAFMSPKDIASQLPTKNPDAHIVLDRILRLLASYSVLNCSL :  95
                  MGSTSSETQISPAQGSDEEANLLAMQLTSASVLPMVLKSAIELDLLEIIAKAGPDAFMSPKDIASQLPTKNPDAHIVLDRILRLLASYSVLNCSL

                       100         *         120         *         140         *         160         *         180         *
Ciclev10020814m : RNLPDGKVERLYGLAPVCKFLTKNEDGVTLSDLCLMNQDKVLMESWYYLKDAVLEGGIPFNKAHGMNAFDYHGKDLRFNKIFNNGMSSHSTITMK : 190
CrOMT2          : RNLPDGKVERLYGLAPVCKFLTKNEDGVTLSDLCLMNQDKVLMESWYYLKDAVLEGGIPFNKAYGMNAFDYHGKDLRFNKIFNNGMSSHSTITMK : 190
ADK97702.1      : RNLPDGKVERLYGLAPVCKFLTKNEDGVTLSDLCLMNQDKVLMESWYYLKDAVLEGGIPFNKAYGMNAFDYHGKDLRFNKIFNNGMSSHSTITMK : 190
                  RNLPDGKVERLYGLAPVCKFLTKNEDGVTLSDLCLMNQDKVLMESWYYLKDAVLEGGIPFNKAyGMNAFDYHGKDLRFNKIFNNGMSSHSTITMK

                          200         *         220         *         240         *         260         *         280
Ciclev10020814m : KILENYKGFEGLNSVVDVGGGIGATLNMIISKYPSIKGINFDLPHVS---------SMLGGDMFVSVPKGDAIFIKWICHDWSDEHCVKFLKNCY : 276
CrOMT2          : KILENYKGFEGLNSVVDVGGGIGATLNMIISKYPSIKGINFDLPHVIQDAPAFPGVEHVGGDMFVSVPKGDAIFIKWICHDWSDEHCVKFLKNCY : 285
ADK97702.1      : KILENYKGFEGLNSVVDVGGGIGATLNMIISKYPSIKGINFDLPHVIQDAPAFPGVEHVGGDMFVSVPKGDAIFIKWICHDWSDEHCVKFLKNCY : 285
                  KILENYKGFEGLNSVVDVGGGIGATLNMIISKYPSIKGINFDLPHViqdapafpgveh6GGDMFVSVPKGDAIFIKWICHDWSDEHCVKFLKNCY

                      *         300         *         320         *         340         *         360
Ciclev10020814m : EALPVNGKVIVAESILPVTPDTSLASKVVIHVDCIMLAHNPGGKERTEQEFRALAKAAGFQGFQVVSSAFNTYIMEFLKSA : 357
CrOMT2          : EALPVNGKVIVAESILPVTPDTSLASKVVIHVDCIMLAHNPGGKERTEQEFRALAKAAGFQGFQVVSSAFNTYIMEFLKSA : 366
ADK97702.1      : EALPVNGKVIVAESILPVTPDTSLASKVVIHVDCIMLAHNPGGKERTEQEFRALAKAAGFQGFQVVSSAFNTYIMEFLKSA : 366
                  EALPVNGKVIVAESILPVTPDTSLASKVVIHVDCIMLAHNPGGKERTEQEFRALAKAAGFQGFQVVSSAFNTYIMEFLKSA
```

C

Percent identity matrix for DNA

	Ciclev10020814m	*CrOMT2*	*HM641694.1*
Ciclev10020814m	100.00	98.79	98.42
CrOMT2		100.00	99.64
HM641694.1			100.00

Percent identity matrix for protein

	Ciclev10020814m	CrOMT2	ADK97702.1
Ciclev10020814m	100.00	98.60	98.60
CrOMT2		100.00	100.00
ADK97702.1			100.00

Figure S1 Sequences alignment of *Citrus reticulata* *O*-methyltransferase 2 (CrOMT2) and two paralogous genes from different citrus species at DNA and protein levels.

Complete CDs (A) and the deduced amino acid sequences (B) of *CrOMT2* and the two paralogous genes were aligned using Clustal2.1 and analyzed by GeneDoc, including *Ciclev10020814m* from *Citrus clementina*, *CrOMT2* from *Citrus reticulata*, and a putative caffeic acid *OMT* from *Citrus aurantium* (GenBank accession: HM641694.1 for complete CDs and ADK97702.1 for the deduced protein). The percent identity matrixes of the aligned sequences created by Clustal2.1 are shown (C).

Figure S2

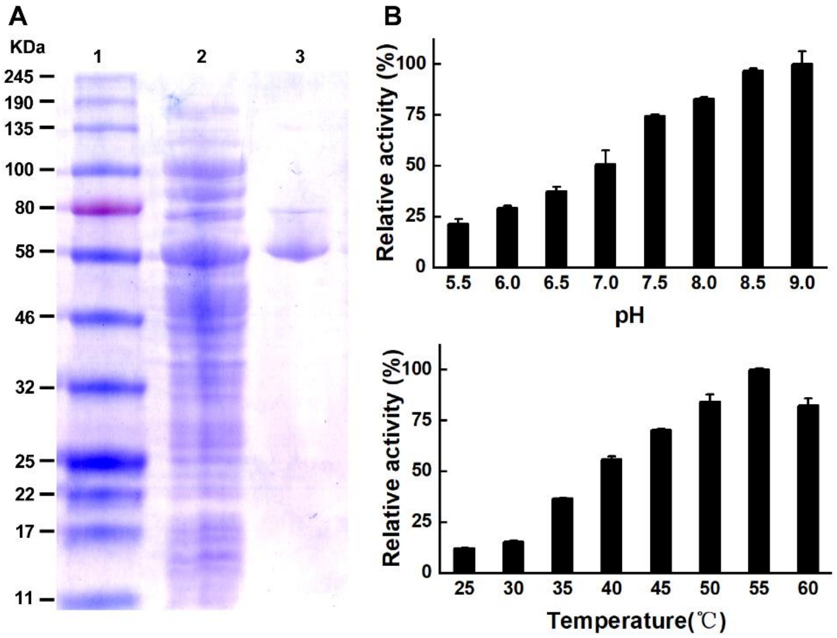

Figure S2 Biochemical characterization of the recombinant CrOMT2.

(A) SDS-PAGE profiles of the recombinant CrOMT2. Lanes: 1, the molecular weight of standard proteins; 2, unpurified fractions after induction; 3, purified enzyme.

(B) The dependency of the enzyme on pH and temperature.

Enzyme activity was measured with luteolin as the substrate, and the corresponding product catalyzed by CrOMT2 was analyzed using HPLC. The data are shown as means ± SE (n = 3).

Figure S3

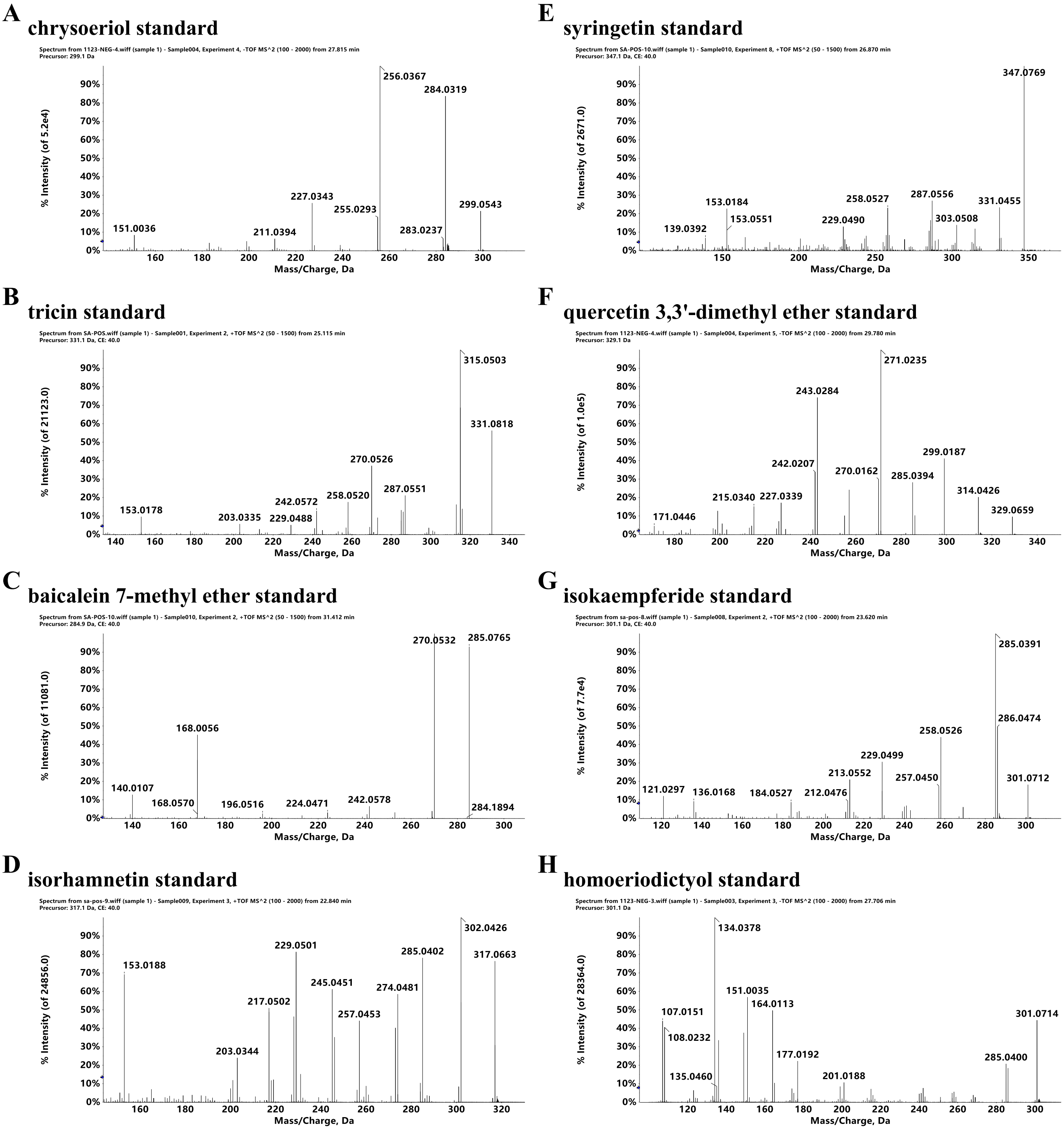

Figure S3 MS/MS spectra of the authentic standards corresponding to the methylated products catalyzed by CrOMT2. Chrysoeriol (A), tricin (B), baicalein 7-methyl ether (C), isorhamnetin (D), syringetin (E), quercetin 3,3'-dimethyl ether (F), isokaempferide (G), homoeriodictyol (H). (A, F, H) were operated in negative ions mode and the rest were in positive ions mode.

Figure S4

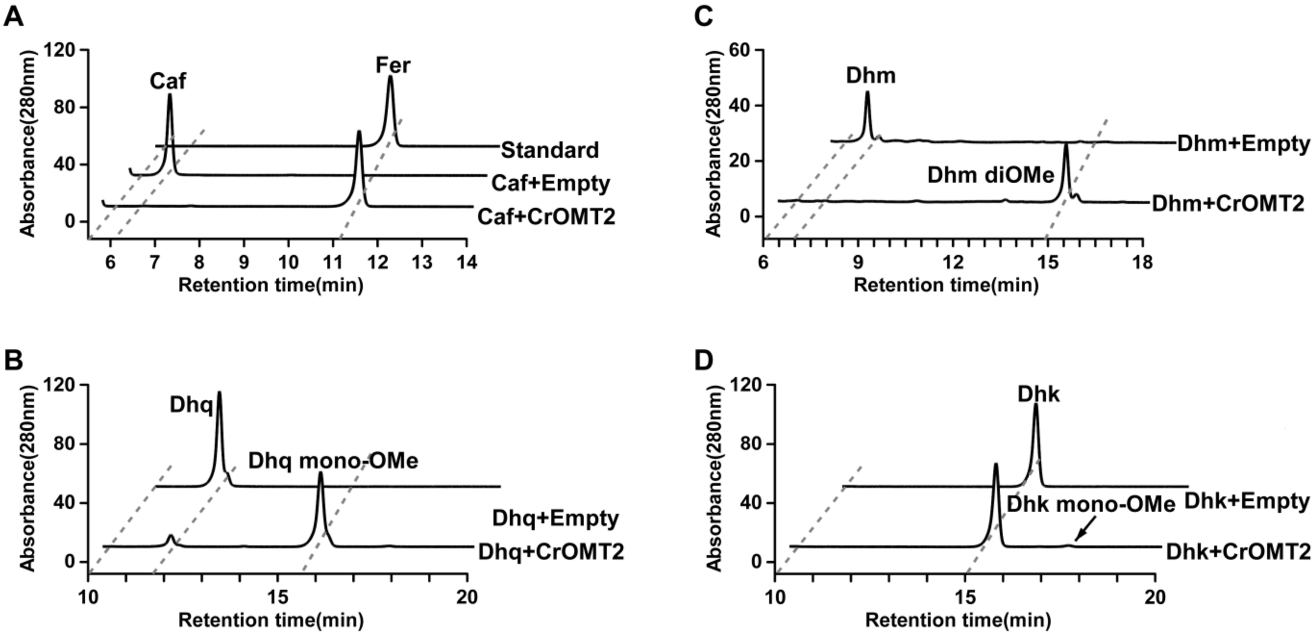

Figure S4 HPLC analysis of dihydroflavonols and caffeic acid methylation by recombinant CrOMT2. HPLC chromatograms of enzyme reactions using caffeic acid (A), dihydroquercetin (B), dihydromyricetin (C) and dihydrokaempferol (D) are shown. The methylated product of caffeic acid produced by the recombinant CrOMT2 were inferred by comparison of the retention times with corresponding standards. For each dihydroflavonols substrate, a single peak generated by CrOMT2 was indicated. Caf, caffeic acid; Fer, ferulic acid; Dhq, dihydroquercetin; Dhm, dihydromyricetin; Dhk, dihydrokaempferol.

Figure S5

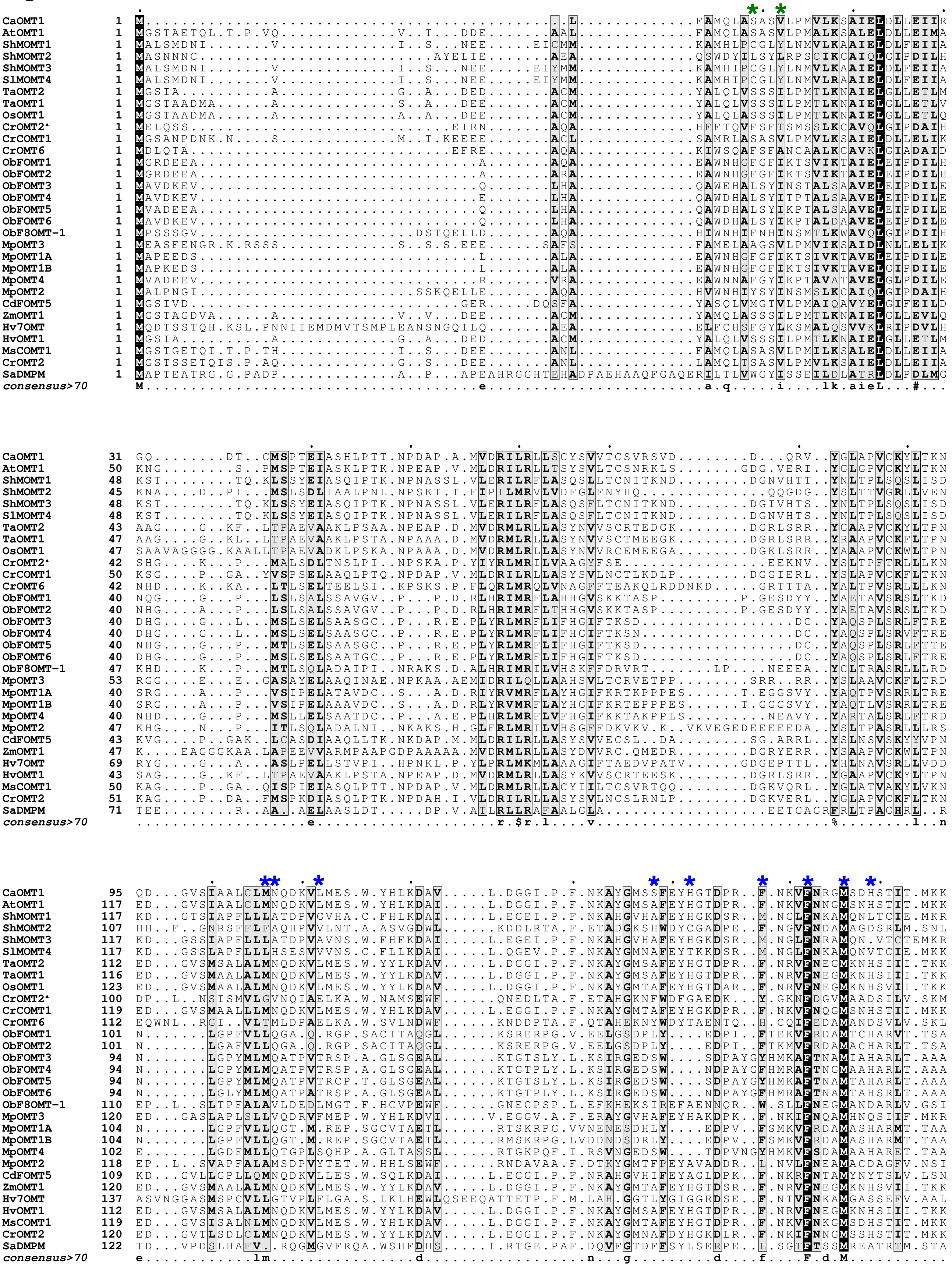
CaOMT1
AtOMT1
ShMOMT1
ShMOMT2
ShMOMT3
SlMOMT4
TaOMT2
TaOMT1
OsOMT1
CrOMT2*
CrCOMT1
CrOMT6
ObFOMT1
ObFOMT2
ObFOMT3
ObFOMT4
ObFOMT5
ObFOMT6
ObF8OMT-1
MpOMT3
MpOMT1A
MpOMT1B
MpOMT4
MpOMT2
CdFOMT5
ZmOMT1
Hv7OMT
HvOMT1
MsCOMT1
CrOMT2
SaDMPM
consensus>70

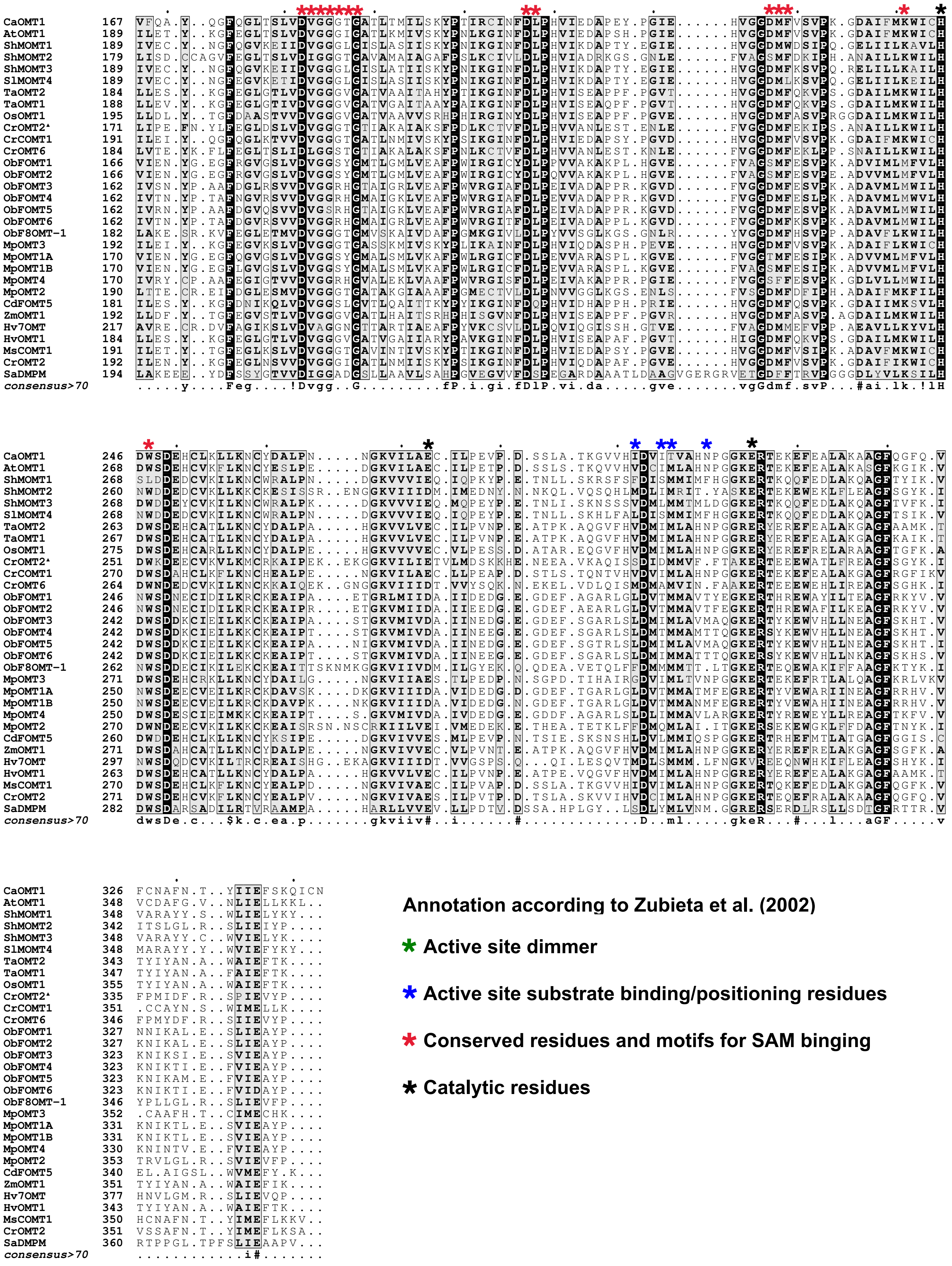
Annotation according to Zubieta et al. (2002)
* Active site dimmer
* Active site substrate binding/positioning residues
* Conserved residues and motifs for SAM binging
* Catalytic residues

Figure S5 Sequence alignment of CrOMT2 and other caffeic acid *O*-methyltransferases (COMTs) from plants.

Protein sequences were aligned via T-Coffee (Expresso) using structural information and analyzed using ESpript 3.0. Residues that are important for dimer formation, substrate specificity, SAM binding and catalysis are shown [29], and are indicated with asterisks in green, blue, red and black, respectively. To avoid ambiguity, CrOMT2 from *Catharanthus roseus* was marked with a triangle.

Figure S6

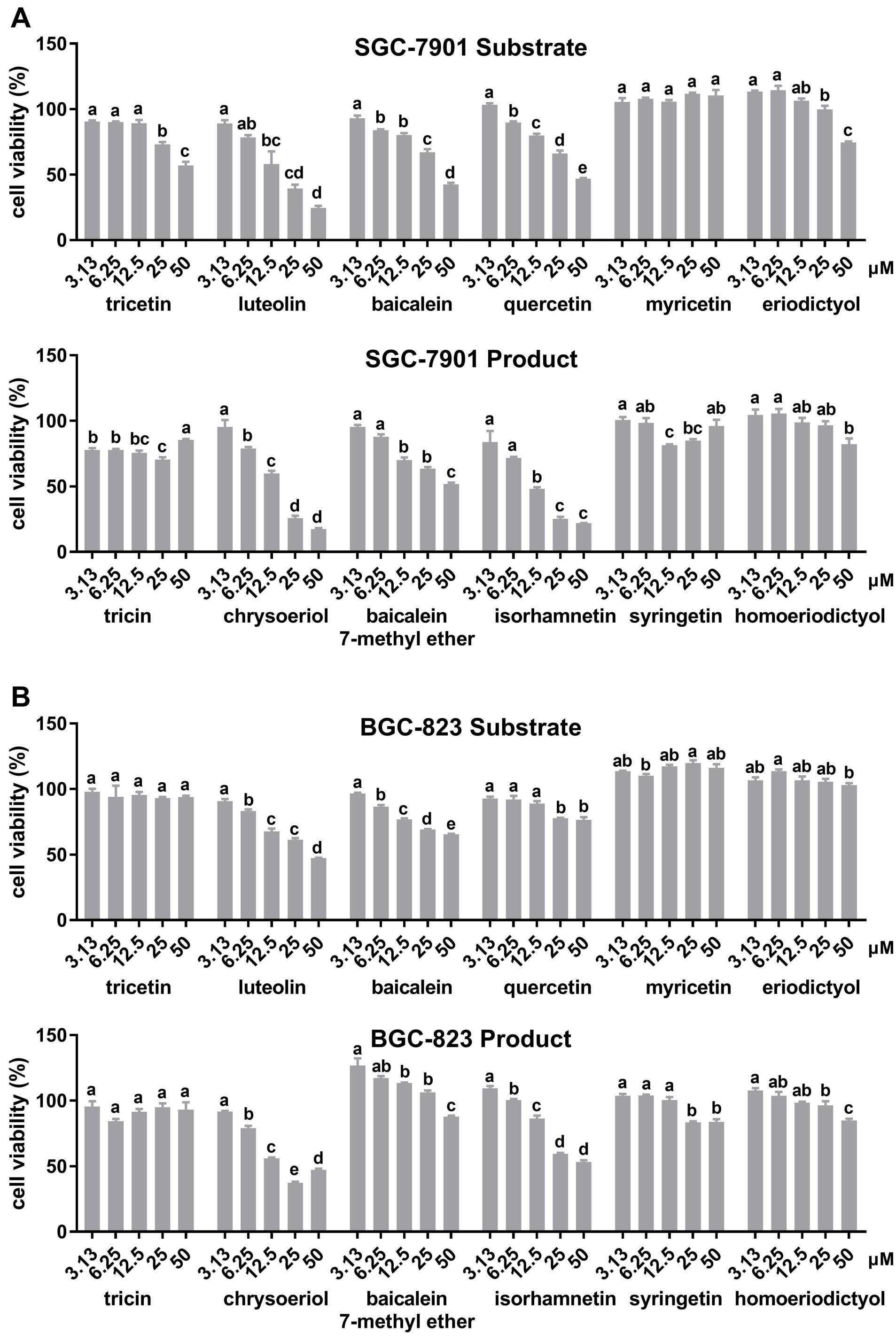

Figure S6 Cell viability after treated with flavonoids substrates or methylated products of CrOMT2 *in vitro*. For each cell line, bars marked by different letters within each index are significantly different ($p < 0.05$) following Tukey's test.

Table S1 Percent identity of AtOMT1 against OMTs in *Citrus clementina*.

Query accesion.version	Subject accession.version	Percentage of identical matches	Expect value
AtOMT1	Ciclev10020814m	73.35	0
AtOMT1	Ciclev10024037m	72.53	0
AtOMT1	Ciclev10005230m	70.67	0
AtOMT1	Ciclev10020761m	70.20	0
AtOMT1	Ciclev10001661m	59.34	1.25E-147
AtOMT1	Ciclev10031952m	57.40	6.45E-141
AtOMT1	Ciclev10018226m	56.79	2.81E-142
AtOMT1	Ciclev10031953m	56.50	6.40E-138
AtOMT1	Ciclev10017683m	56.39	1.41E-142
AtOMT1	Ciclev10031951m	56.19	5.49E-138
AtOMT1	Ciclev10015685m	56.11	3.10E-142
AtOMT1	Ciclev10020875m	56.03	1.97E-142
AtOMT1	Ciclev10031949m	55.89	5.32E-137
AtOMT1	Ciclev10021145m	55.51	7.62E-94
AtOMT1	Ciclev10001555m	55.14	5.37E-154
AtOMT1	Ciclev10032454m	55.07	1.45E-85
AtOMT1	Ciclev10026361m	54.74	9.03E-81
AtOMT1	Ciclev10013542m	54.60	1.06E-127
AtOMT1	Ciclev10020880m	54.60	1.80E-140
AtOMT1	Ciclev10021670m	54.58	9.74E-90
AtOMT1	Ciclev10026359m	54.31	1.45E-80
AtOMT1	Ciclev10017649m	54.10	2.49E-131
AtOMT1	Ciclev10021888m	54.01	9.69E-86
AtOMT1	Ciclev10021678m	53.79	2.35E-90
AtOMT1	Ciclev10032453m	53.74	1.15E-82
AtOMT1	Ciclev10020874m	53.74	1.53E-138
AtOMT1	Ciclev10032027m	53.47	1.03E-124
AtOMT1	Ciclev10012042m	53.43	2.42E-137
AtOMT1	Ciclev10021321m	53.25	2.98E-79
AtOMT1	Ciclev10021326m	53.25	2.98E-79
AtOMT1	Ciclev10023568m	52.87	9.58E-137
AtOMT1	Ciclev10020870m	52.87	7.54E-136
AtOMT1	Ciclev10020873m	52.87	7.54E-136
AtOMT1	Ciclev10032452m	52.86	3.64E-82
AtOMT1	Ciclev10023994m	52.59	2.50E-128
AtOMT1	Ciclev10032460m	52.42	1.19E-80
AtOMT1	Ciclev10020782m	51.62	3.91E-130
AtOMT1	Ciclev10010860m	51.29	3.91E-81
AtOMT1	Ciclev10020818m	50.82	1.05E-130
AtOMT1	Ciclev10020821m	50.29	5.51E-120
AtOMT1	Ciclev10020377m	50.14	6.29E-122
AtOMT1	Ciclev10020820m	50.00	9.12E-121

Query accesion.version	Subject accession.version	Percentage of identical matches	Expect value
AtOMT1	Ciclev10005251m	49.45	8.43E-127
AtOMT1	Ciclev10020828m	49.13	1.75E-119
AtOMT1	Ciclev10023965m	46.49	5.23E-114
AtOMT1	Ciclev10003737m	46.24	2.02E-81
AtOMT1	Ciclev10003323m	43.15	1.69E-87
AtOMT1	Ciclev10012087m	42.07	4.53E-89
AtOMT1	Ciclev10005309m	40.85	2.85E-84
AtOMT1	Ciclev10006704m	39.94	4.03E-78

Table S2 Primers used for amplification.

Gene	Forward primer (5' to 3')	Reverse primer (5' to 3')
CrOMT2-CDs	ATGGGTTCAACCAGTTCAGAAAC	TCAAGCACTCTTGAGAAATTCC
CrOMT2-PET	ATAGGATCCATGGGTTCAACCAG TTCAGAAAC	GAAGGACTCGAGAGCACTCTTG AGAAATTCC

Underlined sequences represent restriction enzyme sites

Table S3 UniProt entries of COMT proteins used in the phylogenetic tree.

Protein names	UniProt entry	Organism
AtOMT1	Q9FK25	*Arabidopsis thaliana* (Mouse-ear cress)
CrOMT2	Q8GSN1	*Catharanthus roseus* (*Madagascar periwinkle*) (Vinca rosea)
CrCOMT1	Q8W013	*Catharanthus roseus* (*Madagascar periwinkle*) (Vinca rosea)
CrOMT6	Q6VCW3	*Catharanthus roseus* (*Madagascar periwinkle*) (Vinca rosea)
CaOMT1	P59049	*Chrysosplenium americanum* (Golden saxifrage)
CdFOMT5	A0A125T1T5	*Citrus depressa*
HvOMT1	Q43771	*Hordeum vulgare* (Barley)
Hv7OMT	A5YTR4	*Hordeum vulgare* subsp. *vulgare* (Domesticated barley)
MsCOMT1	P28002	*Medicago sativa* (Alfalfa)
MpOMT3	Q6VMV9	*Mentha piperita* (Peppermint) (*Mentha aquatica* x *Mentha spicata*)
MpOMT1A	Q6VMW2	*Mentha piperita* (Peppermint) (*Mentha aquatica* x *Mentha spicata*)
MpOMT1B	Q6VMW1	*Mentha piperita* (Peppermint) (*Mentha aquatica* x *Mentha spicata*)
MpOMT4	Q6VMV8	*Mentha piperita* (Peppermint) (*Mentha aquatica* x *Mentha spicata*)
MpOMT2	Q6VMW0	*Mentha piperita* (Peppermint) (*Mentha aquatica* x *Mentha spicata*)
ObFOMT1	K0I977	*Ocimum basilicum* (Sweet basil)
ObFOMT2	K0II72	*Ocimum basilicum* (Sweet basil)
ObFOMT3	K0I7Q2	*Ocimum basilicum* (Sweet basil)
ObFOMT4	K0I210	*Ocimum basilicum* (Sweet basil)
ObFOMT5	K0ICR0	*Ocimum basilicum* (Sweet basil)
ObFOMT6	K0I986	*Ocimum basilicum* (Sweet basil)
ObF8OMT-1	S5DWK8	*Ocimum basilicum* (Sweet basil)
OsOMT1	Q6ZD89	*Oryza sativa* subsp. *japonica* (Rice)
ShMOMT1	F2YTN4	*Solanum habrochaites* (Wild tomato) (*Lycopersicon hirsutum*)
ShMOMT2	F2YTN5	*Solanum habrochaites* (Wild tomato) (*Lycopersicon hirsutum*)
ShMOMT3	M9Z1G5	*Solanum habrochaites* (Wild tomato) (*Lycopersicon hirsutum*)
SlMOMT4	A0A088MF62	*Solanum lycopersicum* (Tomato) (*Lycopersicon esculentum*)
TaOMT2	Q38J50	*Triticum aestivum* (Wheat)
TaOMT1	Q84N28	*Triticum aestivum* (Wheat)
ZmOMT1	Q6VWG3	*Zea mays* (Maize)
SaDMPM	P42712	*Streptomyces alboniger*
